# Species interactions drive the spread of ampicillin resistance in human-associated gut microbiota

**DOI:** 10.1093/emph/eoab020

**Published:** 2021-06-24

**Authors:** Siobhán O’Brien, Michael Baumgartner, Alex R Hall

**Affiliations:** 1 Department of Evolution, Ecology and Behaviour, University of Liverpool, Liverpool L69 7ZB, UK; 2 Department of Environmental Systems Science, Institute of Integrative Biology, ETH Zürich, 8092 Zürich, Switzerland

**Keywords:** evolution, ecology, microbiology, antibiotic resistance, competitive release, microbiome

## Abstract

**Background and objectives:**

Slowing the spread of antimicrobial resistance is urgent if we are to continue treating infectious diseases successfully. There is increasing evidence microbial interactions between and within species are significant drivers of resistance. On one hand, cross-protection by resistant genotypes can shelter susceptible microbes from the adverse effects of antibiotics, reducing the advantage of resistance. On the other hand, antibiotic-mediated killing of susceptible genotypes can alleviate competition and allow resistant strains to thrive (competitive release). Here, by observing interactions both within and between species in microbial communities sampled from humans, we investigate the potential role for cross-protection and competitive release in driving the spread of ampicillin resistance in the ubiquitous gut commensal and opportunistic pathogen *Escherichia coli*.

**Methodology:**

Using anaerobic gut microcosms comprising *E.coli* embedded within gut microbiota sampled from humans, we tested for cross-protection and competitive release both within and between species in response to the clinically important beta-lactam antibiotic ampicillin.

**Results:**

While cross-protection gave an advantage to antibiotic-susceptible *E.coli* in standard laboratory conditions (well-mixed LB medium), competitive release instead drove the spread of antibiotic-resistant *E.coli* in gut microcosms (ampicillin boosted growth of resistant bacteria in the presence of susceptible strains).

**Conclusions and implications:**

Competition between resistant strains and other members of the gut microbiota can restrict the spread of ampicillin resistance. If antibiotic therapy alleviates competition with resident microbes by killing susceptible strains, as here, microbiota-based interventions that restore competition could be a key for slowing the spread of resistance.

**Lay Summary:**

Slowing the spread of global antibiotic resistance is an urgent task. In this paper, we ask how interactions between microbial species drive the spread of resistance. We show that antibiotic killing of susceptible microbes can free up resources for resistant microbes and allow them to thrive. Therefore, we should consider microbes in light of their social interactions to understand the spread of resistance.

## BACKGROUND

Overuse and misuse of antibiotics has led to the rapid emergence of resistance. The spread of resistance depends on the rate at which resistance mechanisms arise [1] and population growth of resistant bacteria in the presence [2] and absence [3] of antibiotics. However, it is increasingly clear that the spread of resistant bacteria also depends on how they interact with other strains or species. For example, bacteria can interact competitively (e.g. for shared resources [4]) or cooperatively (e.g. cross-feeding [5]) and we would expect resistant genotypes to spread faster when antibiotics eliminate competing rather than cooperating strains. There is accumulating evidence that interactions within species can alter the rate and trajectory of antibiotic resistance evolution [6–10]. However, the effects of antibiotic resistance mechanisms are not solely directed at conspecifics, suggesting that inter-specific interactions are also important for driving resistance [11–14]. Nevertheless, our understanding of how antibiotic resistance is shaped by intra- and inter-species interactions in diverse microbial communities (such as the human gastrointestinal tract) remains limited [15, 16]. Given that antibiotic-resistant pathogens exist in complex communities in nature, progress here will facilitate the prediction and management of resistance.

Some types of intra- and inter-specific interactions are likely to slow the spread of resistant genotypes upon exposure to antibiotics. For example, if growth of resistant bacteria reduces the effective antibiotic concentration experienced by susceptible strains or species (cross-protection), the relative fitness advantage of resistance is reduced (compared to in the absence of such cross-protection). This effect has been observed for β-lactam antibiotics degraded by β-lactamase-producing microbes, benefiting not just the producer but nearby susceptible cells. This allows susceptible strains to act as social cheats and gain a frequency-dependent fitness advantage [6, 7, 9, 15, 17, 18]. However, cross-protection is sensitive to factors such as spatial structure [18], which reduces population mixing and opportunities for exploitation. Moreover, in complex communities, such as the mammalian gastrointestinal tract, the potential for cross-protection will depend on which resistance mechanisms are circulating and whether resident strains or species can detoxify the environment in other ways (e.g. inoculum effects [19]). Therefore, while results from simplified systems indicate cross-protection can occur in some scenarios, it is not yet clear whether cross-protection plays a role in protecting susceptible genotypes in complex communities. One recent study [15] found evidence of kanamycin cross-protection in a pig gut microbiota, albeit only at intermediate antibiotic concentrations (2–20 μg/ml).

Conversely, other types of microbial interactions could promote the spread of resistance. For example, when competitive interactions dominate microbial communities, antibiotic therapy can kill sensitive competitors, freeing up resources for resistant strains and driving the spread of resistance through a community or population (competitive release [20]). Competitive release is a driving force behind *Clostridium difficile* infection of the gut, where treatment with broad-spectrum antibiotics kills protective microbiota, opening up niche space for the invasion of *C.difficile* [21]. A similar phenomenon is observed in the cystic fibrosis lung, where loss of microbial community diversity in response to antibiotic treatment in early childhood precedes invasion by the highly antibiotic-resistant pathogen *Pseudomonas aeruginosa* [22–24]. There is also evidence for competitive release driving within-species population dynamics, primarily chemotherapy of acute infections of the rodent malaria *Plasmodium chabaudi* in laboratory mice. In these studies, an expansion in the numbers of resistant parasites is observed following drug administration [25–28].

The relative importance of cross-protection and competitive release in the human gastrointestinal tract is not well understood, even though this is a key battleground in the antibiotic resistance crisis [29]. In part, this knowledge gap reflects the difficulty of quantifying the net effect of interactions with other strains or species in communities sampled from human gastrointestinal tracts. Here, we overcome this challenge using an anaerobic human gut microcosm system [16]. We chose *Escherichiacoli* as our focal strain because it is a ubiquitous gut commensal [30–32] and key opportunistic pathogen with rising antibiotic resistance [33]. We focus on the beta-lactam antibiotic ampicillin, because it is widely used and resistance is a key problem in *E.coli* [30], including via mechanisms that also apply to other species and antibiotics [31]. We inoculated each microcosm with susceptible and/or resistant genotypes of a focal *E.coli* strain, before tracking their population growth with/without ampicillin, and with/without the resident microbiota. By inoculating resistant and sensitive genotypes of our focal strain both in monocultures (where the two genotypes cannot directly compete or engage in cross-protection) and in co-cultures (allowing possibilities for competition and cross-protection), we simultaneously observed opportunities for cross-protection and competitive release, both within and between species. We hypothesized that if cross-protection is in play, the susceptible focal *E.coli* strain would experience weaker ampicillin inhibition in the presence-versus-absence of (i) the resistant focal *E.coli* strain, and/or (ii) the resident microbiota (if the microbiota contains microbes that reduce the effective antibiotic concentration in the microcosm). Conversely, competitive release driven by ampicillin inhibition of sensitive bacteria would result in the resistant focal *E.coli* strain growing better in the presence-versus-absence of (i) the resident microbiota (if the microbiota contains ampicillin-susceptible competitors) and/or (ii) sensitive focal strain. Our results show that while cross-protection increased the relative fitness of antibiotic-susceptible *E.coli* in standard laboratory conditions [well-mixed lysogeny broth (LB)], competitive release of antibiotic-resistant *E.coli* drove the spread of resistance in human gut microcosms.

## METHODOLOGY

### Bacterial strains

We used *E.coli* K-12 MG1655 with a chromosomal fluorescent dTomato tag and a chloramphenicol resistance cassette as our ampicillin-susceptible strain (K-12_susc_). For our ampicillin-resistant strain (K-12_res_), we inserted a non-conjugative *bla*_TEM_ plasmid conferring ampicillin resistance into K-12_susc_ [34] (Supplementary methods S1). We confirmed the stability of the plasmid by verifying that the number of K-12_res_ colonies did not differ between LB plates either supplemented with or without 100 μg/ml ampicillin after 24h growth in LB media (mean counts in LB medium: 84.3 ± 6.8; mean CFU counts LB supplemented with ampicillin: 88.7 ± 9.1). We note *E.coli* K-12 possesses an inducible ampC beta-lactamase [35], which may influence its susceptibility to ampicillin; there was nevertheless a clear difference in susceptibility between our susceptible and resistant focal strains here. Resistance to chloramphenicol ensured both strains could be isolated from the microbiota when plated on LB agar supplemented with 25 μg/ml chloramphenicol.

### Human microbiome samples

We collected stool samples from three human donors on 15 May 2018. Samples were stored at −80°C until this experiment was conducted in July 2019 (Supplementary methods S1). The full sampling regime is outlined in Reference [16] and approved by the ETH Zürich Ethics Commission (EK 2016-N-55). For our competition experiment in anaerobic gut microcosms, we combined all three samples and tested how the fitness of K-12_res_ and K-12_susc_ depended on the presence or absence of this combined gut microbiota. We used frozen stool samples to make faecal slurry, consistent with our aim of including microbial communities sampled from human gastrointestinal tracts (but not necessarily reproducing entire communities or all physiological conditions, which is unrealistic *ex vivo*). Past work indicates frozen samples are taxonomically similar to fresh samples and have similar effects in downstream experiments in anaerobic fermenters [36]. We chose to pool the microbiota samples from the three donors for three reasons. First, this allowed us to test the effects of multiple factors (microbiota, ampicillin and culture conditions) with multiple replicates in each treatment and extending the generality of our results beyond a single human donor sample, but with a feasible experimental scale. Second, pooled samples have been used successfully in experiments with anaerobic fermenters filled with human gut slurry [37]. Third, previous amplicon sequencing showed that taxonomic composition of these three sampled communities was similar (dominated by *Ruminococcaceae*, *Lachnospiraceae* and *Bacteroidaceae*), suggesting that pooling them results in an aggregate microbiota, rather than an entirely novel kind of community [16]. The final combined slurry was plated on LB agar supplemented with 25 μg/ml chloramphenicol to ensure the culturable component of the resident microbiota was susceptible to this antibiotic. This facilitated the isolation of our chloramphenicol-resistant focal *E.coli* strains in our competition experiments. Finally, we confirmed that the culturable component of our microbiota was inhibited by ampicillin (total colony counts were approximately halved when plated on LB agar supplemented with 100 μg/ml ampicillin compared to ampicillin-free plates). This indicated the presence of both ampicillin-resistant and susceptible microbes within the microbiota, and therefore potential opportunities for microbiota-mediated cross-protection or competitive release, respectively. This was further confirmed using flow cytometry in our anaerobic gut microcosm experiment (below).

### Competition assays in LB media

We first established whether ampicillin resistance in our focal strain could confer a protective benefit to susceptible cells in well-mixed, nutrient-rich, standard laboratory media, exposed to sublethal ampicillin concentrations (where we expected cross-protection to be relatively likely [38]). To test this, we grew K-12_res_ and K-12_susc_ alone (monoculture) and together (co-culture) in the presence and absence of 7.2 μg/ml ampicillin (∼90% of the minimum inhibitory concentration, of the sensitive strain [16]). For monocultures, we added ∼10^5^cfu/ml each strain to 5 ml LB (Sigma-Aldrich). For co-cultures, we added ∼5 × 10^4^ cfu/ml each strain, so the total density was similar to monocultures. We made six replicates for each set of conditions (36 cultures in total). We then incubated each culture at 37°C, shaking at 180 rpm for 24 h, before serially diluting and plating on (i) LB agar (Sigma-Aldrich) and (ii) LB agar supplemented with ampicillin (100 μg/ml). This allowed us to enumerate total bacterial density and that of K-12_res_ only. K-12_susc_ densities were estimated by subtracting K-12_res_ from total bacterial densities.

We estimated the total change in population density for each strain in each microcosm as the Malthusian growth parameter (*m*): ln(final density/start density) [39]. Starting densities were quantified by plating overnight cultures of each strain before inoculation. We then tested whether ampicillin (with vs without) and/or culture conditions (monoculture vs co-culture) affected the growth (*m*) of each strain (K-12_res_ and K-12_susc_) using analysis of variance, fitted separately for each strain and including the ampicillin×culture conditions interaction. We used Box–Cox transformation to improve the normality of the data, after first adding a constant value (1.5) to all *m* values (accounting for four cases, where *m* ≤ 0; further details are given below). Thus, in both the presence and absence of antibiotics, we tested whether growth of each strain (K-12_susc_ and K-12_res_) was higher or lower when grown in isolation (monoculture) or together (co-culture), following earlier work on social interactions among closely related strains [40, 41]. We used population growth (*m*), rather than final population density (cfu/ml), as our response variable here because this accounts for variation in starting densities of each strain (e.g. in co-cultures relative to monocultures). Thus, if each strain grows equally well (same number of replications, and similar values of *m*) in monoculture versus co-culture, this suggests cells of each type replicate similarly well when surrounded by clonemates (monoculture) as when surrounded by a mixture of clonemates and cells of the other type (co-culture). We also provide the final population densities (cfu/ml) from these experiments (Supplementary Fig. S1), which supported similar qualitative conclusions. Data were analysed using R version 3.2.4.

### Competition assays in anaerobic gut microcosms

A total of 36 Hungate tubes were filled with 7 ml basal medium (2 g/l peptone, 2 g/l tryptone, 2 g/l yeast extract, 0.1 g/l NaCl, 0.04 g K_2_HPO_4_, 0.04 g/l KH_2_PO_4_, 0.01 g/l MgSO_4_7H_2_O, 0.0 g/l CaCl_2_6H_2_O, 2 g/l NaHCO_3_, 2 ml tween 80, 0.005 g/l haemin, 0.5 g/l L-cysteine, 0.5 g/l bile salts, 2 g/l starch, 1.5 g/l casein, 0.001 g/l resazurin, pH adjusted to 7, addition of 0.001 g/l menadion after autoclaving; Sigma-Aldrich). The headspace of each tube was flushed with nitrogen gas and sealed with a rubber septum, before autoclaving.

**Figure 1.**
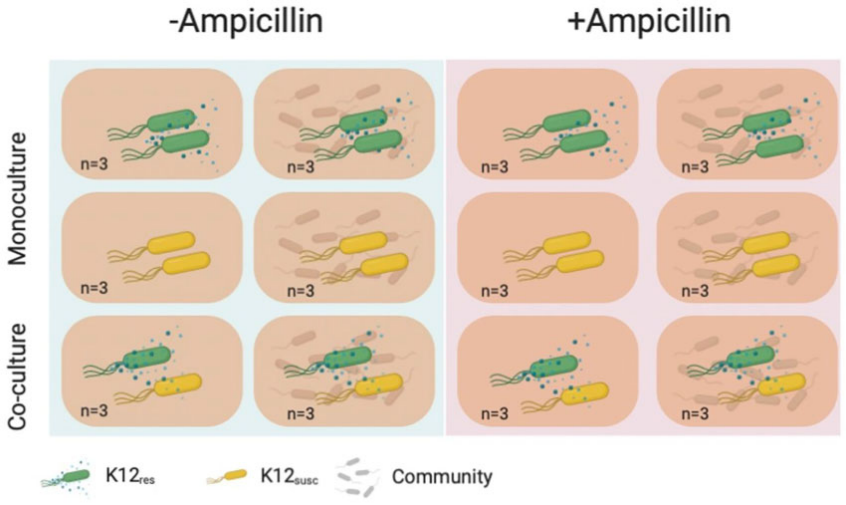
Experimental design to test how the resident microbial community, ampicillin and culture condition (monoculture vs co-culture) interact to influence the growth of resistant and susceptible genotypes (K-12res and K-12sus) of our focal *E.coli* strain

We inoculated each tube with (i) 10^5^ cfu/ml K-12_res_ (ii) 10^5^ cfu/ml K-12_susc_ or (iii) 5 × 10^4^ cfu/ml of each, as above. Half of the tubes (*n* = 18) were exposed to 7.2 μg/ml ampicillin and the remaining half with an equivalent volume of sterilized water. Finally, half of our tubes (*n* = 18) were inoculated with the resident microbiota (350 μl of ‘fresh’ gut slurry plus 500 μl of sterilized slurry) and the remaining half with a microbiota-free control (850 μl sterilized gut slurry), giving a total volume in each tube of 8 ml. Each treatment combination was replicated thrice, giving a fully factorial experimental design (Fig. 1).

Tubes were grown without shaking at 37°C for 24 h, before diluting and plating on (i) LB agar supplemented with 25 μg/ml chloramphenicol (to distinguish the focal strain from resident microbiota) and (ii) LB agar supplemented with 25 μg/ml chloramphenicol and 100 μg/ml ampicillin (to distinguish K-12_res_ from K-12_susc_). Under this plating regime, the detection limit for the final abundance of K-12_susc_ is high when K-12_susc_ is very rare relative to K-12_res_. In these cases (where the count from the ampicillin plate was equal to or greater than the count from the ampicillin-free plate), we assigned a final K-12_susc_ density equal to its starting density in the same microcosm (that is, assuming zero net population growth in these microcosms, *n* = 3; these cases have *m* = 0 in Fig. 3). We verified that colonies on our selective plates were derived from the focal strain (and not resident *E.coli*) by using a fluorescence-capable stereoscope, confirming that all plated colonies had the dTomato marker. Finally, to quantify ampicillin inhibition of the microbial community in each microcosm, we measured total microbial abundance (cells/ml) using flow cytometry (benchtop flow cytometer Novocyte 2000 R, ACEA Biosciences Inc.), described in Reference [16]. This procedure separates cells from background noise in our system, as elsewhere [42] although we acknowledge flow cytometry can have other limitations (e.g. detecting cells in aggregates or clumps).

As above in LB, we estimated the total change in population density for each variant of the focal strain (K-12_susc_ and K-12_res_) in each microcosm as the Malthusian growth parameter (*m*). We then tested whether ampicillin (with vs without), culture conditions (monoculture vs co-culture) and resident microbiota (with vs without) affected the growth (*m*) of each strain (K-12_res_ and K-12_susc_) using analysis of variance, fitted separately for K-12_sus_ and K-12_res_, and including interaction terms. Thus, interactions between K-12_res_ and K-12_susc_ were tested as above in LB medium, but interactions of each of these strains with the resident microbiota were tested in a slightly different way. Specifically, by this experimental design, the total initial focal strain population density (in both mono- and co-cultures) was the same in microcosms incubated with versus without the resident microbiota. This allowed us to test whether addition of the resident microbiota competitively suppressed the focal strain, by comparing focal strain growth with versus without resident microbiota [43, 44]. As with our LB experiment, we also provide final densities (cfu/ml) from these experiments, which support the same qualitative conclusions (Supplementary Figs S2 and S3).

### Supernatant addition experiment

Some key mechanisms of competition involve changes to the local abiotic environment (e.g. resource depletion or accumulation of toxins). We therefore tested whether population growth of focal resistant *E.coli* varied upon exposure to supernatants extracted from cultures including (i) focal *E.coli*, (ii) the resident microbiota or (iii) no bacteria. Our goal here was to observe overall differences among these three main classes of supernatants, but to be consistent with the community treatments used in our main anaerobic microcosm experiment (above), we included multiple subtypes within these categories (e.g. resident microbiota alone as well as microbiota in which a focal *E.coli* strain was embedded). Supernatant was obtained by inoculating microcosms of basal medium containing 7.2 μg/ml ampicillin with: (i) 10^5^ cfu/ml K-12_res_, (ii) 10^5^ cfu/ml K-12_susc_, (iii) 350 μl faecal slurry, (iv) 350 μl faecal slurry + 10^5^ cfu/ml K-12_res_ or (v) 350 μl faecal slurry + 10^5^ cfu/ml K-12_susc_. We also included two control tubes containing sterile basal medium. One control tube was treated with ampicillin, the second was not. Cultures were grown for 24 h static at 37°C under anaerobic conditions, after which they were transferred to 15 ml falcon tubes and centrifuged for 5 min at 4000 rpm. The supernatant was removed, and filter sterilized (0.22 filter). We then tested for population growth of K-12_res_ in each supernatant, by inoculating 10^5^ cfu/ml of K-12_res_ into replicate microplate wells containing 198 μl of supernatant (four replicates per treatment). We incubated the microplate at 37°C static for 24 h under aerobic conditions, after which cell densities were quantified using flow cytometry [16].

## RESULTS

### Ampicillin resistance is a shareable public good in well-mixed LB media

We first examined the costs and benefits of ampicillin resistance by measuring the change in population density (Malthusian growth parameter, *m*) for our focal *E.coli* susceptible and resistant strains (K-12_susc_ and K-12_res_), growing under mono- and co-culture conditions, in the presence and absence of ampicillin, in well-mixed LB media.

**Figure 2.**
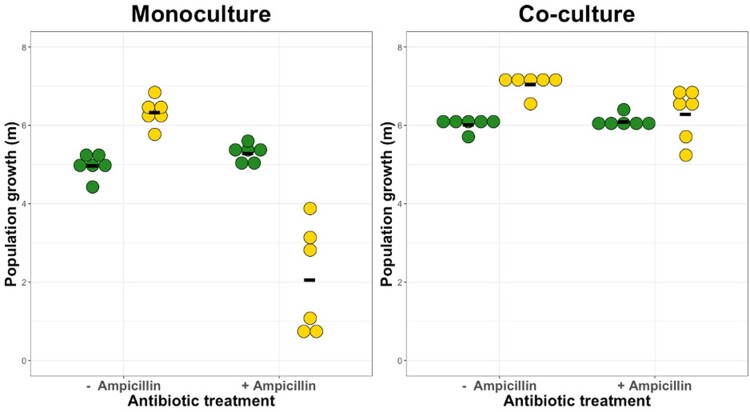
Population growth (*m*) expressed as the Malthusian growth parameter (natural logarithm of the increase in abundance over time, see Methodology) of susceptible (yellow) and resistant (green) genotypes of our focal *E.coli* strain grown in the presence and absence of ampicillin in monoculture (left panel) or co-culture (right panel) conditions. There was a significant interaction between ampicillin and culture conditions, so that ampicillin inhibition of K-12susc was weaker in co-culture versus monoculture—consistent with cross-protection (see main text for statistics). Black horizontal bars show mean values; *n*=6

We found that when both strains grew in separate microcosms, K-12_susc_ grew better than K-12_res_ in the absence of antibiotics (monocultures; Fig. 2; bootstrapped *t*-test, *T*_10_ = 7.14, *P* < 0.001), indicating a resistance cost in this context (equivalent to a reduction in final population density of ∼40%; Supplementary Fig. S1). Exposure to ampicillin reversed this effect: K-12_res_ grew better than K-12_susc,_ indicating a benefit to resistance in the presence of ampicillin (monocultures; Fig. 2; bootstrapped *t*-test, *t* = 5.65, d*f* = 5.3, *P* < 0.03). By contrast, when both strains were in the same microcosm (co-culture), addition of ampicillin had a much weaker inhibitory effect on K-12_susc_ compared to the inhibition observed in monoculture [linear model with Box–Cox transformation (*λ* = 2); culture condition×ampicillin interaction, *F*_1,2__0_ = 26.58, *P* < 0.0001] (Fig. 2). In other words, K-12_susc_ gained a protective benefit from the presence of the resistant strain, consistent with this strain detoxifying the local environment and conferring cross-protection.

Conversely, we found no evidence for competitive release of K-12_res_ via killing of susceptibles on exposure to ampicillin [linear model with Box–Cox transformation (*λ* = 4), culture condition×ampicillin interaction, *F*_1,2__1_ = 0.86, *P* = 0.36] (Fig. 2). Instead, K-12_res_ increased growth on average in co-culture versus monoculture (but this was irrespective of ampicillin addition) [linear model with Box–Cox transformation (λ = 4), culture condition, *F*_1,2__2_ = 34.08, *P* < 0.001] (Fig. 2). Ampicillin addition also increased K-12_res_ growth on average (consistent with a cost of ampicillin resistance reported above), but the effect was very weak [linear model with Box–Cox transformation (*λ* = 4), effect of ampicillin, *F*_1,2__2_ = 4.41, *P* = 0.048] (Fig. 2).

### No evidence for cooperative ampicillin resistance in anaerobic gut microcosms

**Figure 3.**
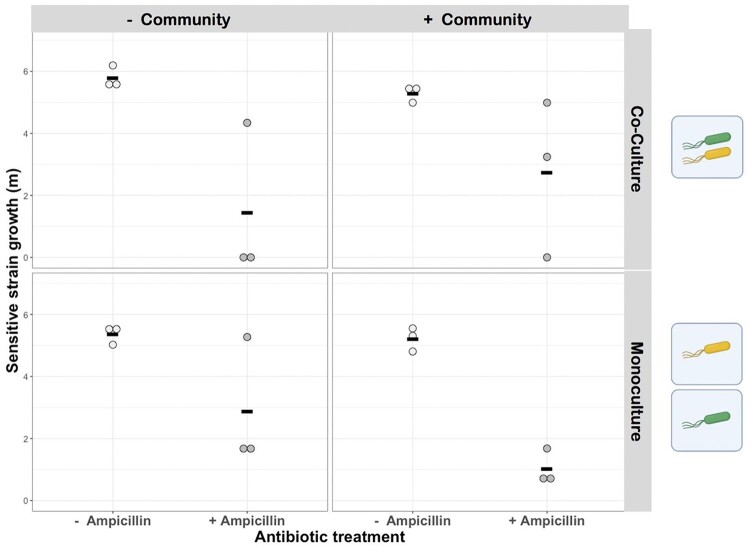
Effects of an antibiotic, an isogenic resistant strain, and a natural microbial community on growth of antibiotic-sensitive *E.coli.* Population growth (*m*) for sensitive *E.coli* (K-12susc) is shown after incubation in the presence and absence of ampicillin (*x*-axis), in the presence and absence (upper vs lower row of panels) of a resistant variant of the same strain (K-12res), and in the presence and absence (right vs left column of panels) of the natural microbial community sampled from human gastrointestinal microbiomes. Ampicillin had a significant effect on K-12susc growth, but neither the resistant strain nor the microbial community did (see main text for statistics). Black horizontal bars show means, *n*=3

In anaerobic gut microcosms (see Methodology), K-12_susc_ growth (*m*) was unaffected by the presence of the resistant strain or the presence of the resident microbiota [linear model with Box–Cox transformation (*λ* = 2); effect of microbiota, *F*_1,2__0_ = 0.55, *P *=* *0.5; effect of culture condition, *F*_1,2__0_ = 0.45, *P* = 0.51] (Fig. 3) over the timescale of our experiment. The strongest effect, we observed on K-12_susc_ growth was for addition of ampicillin, which significantly reduced growth of K-12_susc_ in both mono- and co-cultures and in the presence and absence of resident microbiota [linear model with Box–Cox transformation (*λ* = 2), effect of ampicillin; *F*_1,2__0_ = 40.98, *P* < 0.0001] (Fig. 3). Importantly, we found no evidence that the extent of ampicillin inhibition depended on culture conditions or microbiota treatment [linear model with Box–Cox transformation (*λ* = 2); culture condition×ampicillin interaction, *F*_1,1__7_ = 0.0004, *P* = 0.98; microbiota×ampicillin interaction, *F*_1,1__7_ = 0.04, *P* = 0.85] (Fig. 3). Hence, extracellular detoxification of ampicillin by K-12_res_ or by resident microbiota could not rescue the poor growth of K-12_susc_ in anaerobic gut microcosms.

### Evidence for competitive release in anaerobic gut microcosms

**Figure 4.**
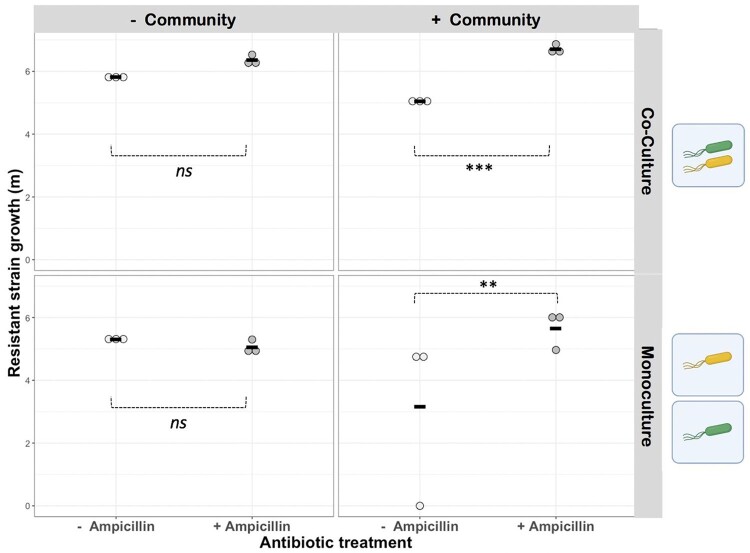
Effects of an antibiotic, an isogenic sensitive strain and a natural microbial community on growth of antibiotic-resistant *E.coli.* Population growth (*m*) for K-12res is shown after growth in the presence and absence of ampicillin (*x*-axis), in the presence and absence of the sensitive K-12sus strain (upper vs lower row of panels) and in the presence and absence of the natural gut microbial community (right vs left columns of panels). Growth (*m*) of K-12res was increased by addition of ampicillin, but only in the presence of the resident microbial community or K-12susc (see main text for statistics). Black horizontal bars show mean values, *n*=3. Asterisks denote *P*<0.05 based on Tukey HSD *post hoc* tests

Unlike for the susceptible K-12_susc_ strain, population growth (*m*) of the resistant K-12_res_ strain was increased by addition of ampicillin, but only in the presence of the resident microbiota [linear model with Box–Cox transformation (*λ* = 4); microbiota×ampicillin interaction: *F*_1,1__7_ = 24.42, *P* = 0.0001; *posthoc* Tukey HSD: effect of ampicillin in treatment groups with microbiota: *P* < 0.05/without microbiota: *P* > 0.05] (Fig. 4). Growth of K-12_res_ was also higher in co-culture with K-12_susc_ compared to monoculture, and this difference was amplified in the presence of ampicillin [linear model with Box–Cox transformation (*λ* = 4); culture condition×ampicillin interaction: *F*_1,1__7_ = 8.99, *P* < 0.01]. Together, this is consistent with ampicillin-mediated killing of susceptible microbes (either our K-12_susc_ strain or susceptible members of the microbiota), that releases K-12_res_ from competition with susceptibles and enhances the spread of ampicillin resistance.

### Competition between resistant bacteria and resident microbiota

If the growth increase observed for resistant bacteria (upon addition of ampicillin in the presence of resident microbiota) were due to competitive release, we would expect K-12_res_ to reach lower densities in the presence versus absence of the resident microbiota without ampicillin (consistent with competitive suppression, from which they can be released by the addition of antibiotics). This is supported by K-12_res_ reaching lower final cell densities in cultures with versus without resident microbiota in the absence of ampicillin (and without the sensitive strain) (linear model, effect of microbiota on final population density: *F*_1,4_ = 10.27, *P* < 0.05). When we take population growth as the Malthusian parameter (instead of final cell densities), we see a similar pattern (lower in every replicate with resident microbiota compared to corresponding replicates without resident microbiota in the absence of ampicillin and without the sensitive strain), although here the effect is not significant on average (Tukey test *P* > 0.05) (Fig. 4). This lack of statistical significance may reflect limited power of this test, dictated by our sample size and detection limit: there was one replicate in the +ampicillin/+microbiota/monoculture treatment where the resistant focal strain was below the detection limit imposed by the plating scheme we used, increasing the within-group variance here. Thus, these data are consistent with competition between K-12_res_ and resident microbes, although they do not demonstrate it conclusively.

**Figure 5.**
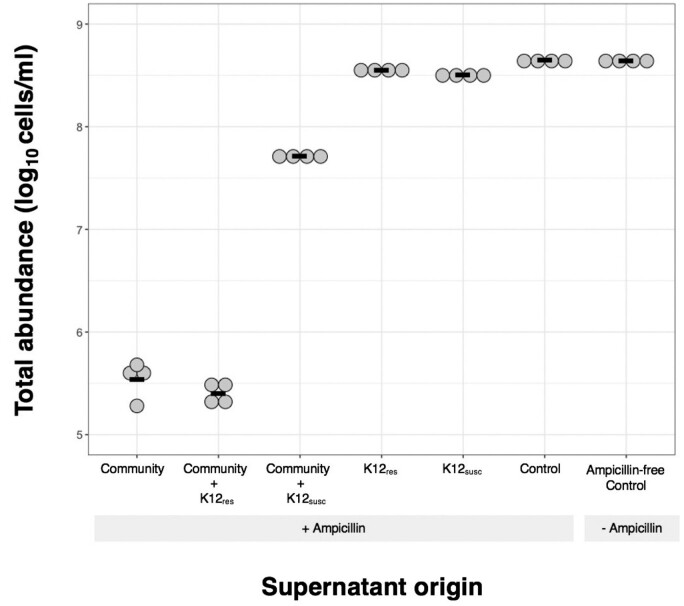
Growth of resistant *E.coli* in supernatant from cultures of sensitive *E.coli* and the natural gut microbial community. Final population density [log10(cells/ml)] for resistant *E.coli* (K-12res) incubated for 24 h in supernatant originating from ampicillin-treated microcosms with or without the resident microbial community, and in bacteria-free controls. Different types of supernatants varied in their ability to support K-12res growth, with supernatants from microbiota treatments consistently supporting less growth than supernatants from *E.coli* cultures, which in turn supported less growth than sterile media (see main text for statistics). Each point shows the mean of four technical replicates for a single population. Black horizontal bars show mean values

As a second test for competition between resident microbiota and the resistant strain, we analysed population growth (*m)* of K-12_res_ in supernatants extracted from cultures with versus without the microbiota (Fig. 5). Different types of supernatants varied in their ability to support K-12_res_ growth (*F*_6,21_ = 476.99, *P* < 0.0001), with supernatants from microbiota treatments consistently supporting less growth than supernatants from cultures containing only a focal *E.coli* strain (Fig. 5). This is consistent with the resident microbiota changing the local abiotic conditions in a way that reduces population growth of K-12_res_ (e.g. via nutrient depletion). Supernatant originating from cultures of focal *E.coli* also supported less growth than supernatant from sterile control tubes (Dunnett’s test; control vs K-12_res_ supernatant, *P* < 0.0001, control vs K-12_susc_ supernatant, *P* < 0.0001).

### Susceptibility of resident microbiota to antibiotic inhibition

A further requirement for competitive release is that addition of ampicillin inhibits the resident microbiota (thereby relieving the negative effect of the microbiota on the resistant strain). Consistent with this, flow cytometric measurements indicated that addition of ampicillin to the resident microbiota had a negative effect on total abundance [linear model with Box–Cox transformation (*λ* = 2), *F*_1,1__4_ = 43.29, *P* < 0.0001, Supplementary Fig. S4]. In particular, in microcosms containing K-12_res_ plus resident microbiota (where we previously observed no inhibition of K-12_res_), the decrease in total abundance here must reflect inhibition of the resident microbiota (Supplementary Fig. S4). Thus, our data support competition between the resistant strain and the resident microbiota, which was alleviated by ampicillin because this inhibited the resident microbiota but not K-12_res._

## CONCLUSIONS AND IMPLICATIONS

Microbes live in complex communities, where species interact both antagonistically (e.g. resource competition or direct killing) as well as cooperatively (e.g. cross-protection or cross-feeding). Understanding how microbes within complex communities, such as the gut microbiota respond to antibiotic treatment is therefore challenging, yet a task that is crucial to predict the spread of antibiotic-resistant pathogens. Here, we provide evidence that in microbial communities sampled from healthy humans, exposure to ampicillin can release a focal resistant *E.coli* strain (K-12_res_) from competition with susceptible bacteria. Growth assays in supernatant indicate this arises from competition between K-12_res_ and the resident microbiota, which is alleviated when ampicillin-susceptible members of the microbiota are killed. Surprisingly, we found no evidence of cross-protection (where susceptible cells benefit from antibiotic-degrading activity of resistant cells) in our gut microcosms, in contrast to results from well-mixed LB medium (this study) and previous work in anaerobic fermenters [15] and *in vivo* [8]. Hence, although cross-protective effects can occur between resistant and susceptible genotypes, they are highly context dependent.

In our experimental setup, competitive release of K-12_res_ was observed in the presence of the resident microbiota upon antibiotic exposure. This is important because it goes beyond past work with other types of pathogens and in non-human microbiota [15, 28] to demonstrate directly that competitive release can contribute to the spread of antibiotic-resistant bacteria in human-associated microbiota. By contrast, while we saw some evidence of competitive release of K-12_res_ in the presence of K-12_susc_ only (i.e. in co-culture, without the microbiota), this effect was much weaker than in the presence of the microbiota. We pose two mutually non-exclusive explanations. First, competitive inhibition of our resistant focal strain by the sensitive focal strain was weaker than inhibition by the resident microbial community. This is supported by our supernatant experiment, where supernatant originating from microcosms containing the resident microbial community supported less growth than supernatant originating from a focal *E.coli* strain only. Possible drivers of this relatively strong suppression by the resident microbiota include resource competition (effective scavenging by a diverse community, or closely related strains that are strong competitors against K-12, as we have seen previously [16]) and/or direct antagonistic interactions, such as toxin production [45]. Second, our experimental design ensured total inoculant densities of our focal *E.coli* strain were consistent between mono- and co-culture conditions. In contrast, the effect of the microbiota was tested by the addition of microbiota to a fixed number of *E.coli* cells. Hence, microbiota-mediated competition for nutrients was directly imposed, whereas competition for nutrients (at least initially) did not differ between co-cultures and monocultures. Note, supernatant from microbiota-free focal strain cultures supported less growth than control supernatant, indicating intra-species competition, albeit with a smaller effect than in microbiota treatments. One caveat is that in order to use flow cytometry to enumerate K-12_res_ cells, supernatant effects were not tested under the same anaerobic conditions established in our gut microcosms (although the supernatant itself was anaerobically produced).

Differences between supernatant subtypes in supporting K-12_res_ growth are harder explain—such as our finding that supernatant from communities in which a susceptible or resistant *E.coli* strain is already embedded differ in their ability to facilitate K-12_res_ growth. One possibility is that killing of K-12_susc_ provides recyclable nutrients that can promote K-12_res_ growth [50]. However, it was beyond the scope of this study to infer what was driving these differences and comparisons between subtypes are supported by low statistical power.

A key insight from our results is that the impact of interactions (both within and between species) on the spread of antibiotic resistance can differ greatly between simple, two-strain experiments and more complex communities. Our experiment in well-mixed LB medium showed that under a specific set of conditions that favour cross-protection [38, 40, 41] ampicillin resistance can be a cooperative public good, increasing the fitness of non-detoxifying ‘cheaters’ that benefit from cross-protection. However, in our gut microcosms, any opportunity for cooperation was trounced by a net competitive interaction between strains. One explanation for these differing dynamics is the relatively high levels of spatial structure in the latter. Spatial structure keeps producers and products closer together, limiting opportunities for exploitation and keeping relatedness between interacting partners high [46]. Note, that localized cross-protection can nevertheless emerge in spatially structured populations, such as those on agar surfaces [47]. Ultimately, the relative importance of cross-protection and competitive release in natural systems will be governed by the balance between resource competition between and within species as well as environmental constraints on cooperation.

Our experiment has some important limitations. Although ampicillin and *E.coli* are undoubtedly of high real-world relevance, generalizing our results to other species/antibiotics should be done with caution. A recent study by Letten *et al.* [48] with a similar setup, but different antibiotics, samples and focal strain, found little evidence that antibiotics released an invading strain from competition with resident microbiota. This difference may be explained by different antibiotics having different effects on resident microbiota, variation among focal resistant strains, or other differences between experiments (e.g. baseline competition with resident microbiota was relatively strong in Letten *et al.* [48]). Conversely, a past experiment also using this study system showed an ampicillin-resistance plasmid conferred a relatively large fitness benefit to its host when embedded in a gut microbiota and exposed to ampicillin [16], consistent with our results here. Together, these findings are in line with the above evidence that competitive release and cross-protection can be important, but identifying the conditions that give rise to them is a key challenge for translation to real-world applications. A second, related limitation is that the dynamics of competitive release we observed may be different with other antibiotic concentrations (we would expect stronger release at higher doses) and over other timescales (past work suggested stronger competitive suppression over longer timescales than in our experiment [16, 49]).

Both in our study, and in a previous study on *P.chabaudi*-infected mice [25] the growth increase of resistant pathogens triggered by competitive release increased beyond that achieved when a competitor had never been present. Wargo *et al.* [25] suggested the mechanism driving this effect is a delayed immune response to an emerging resistant clone under drug exposure. Our experimental setup has no immune system, so this phenomenon is likely here caused by dynamics within the microbial community itself. One possibility is that viable cells can recycle nutrients from the carcases of antibiotic-exposed susceptible cells [50] and this would be an interesting avenue for future work. Crucially, if competitive release can enhance growth above what the pathogen could achieve in isolation, it raises important questions about the indirect impact antibiotic therapy could be having on promoting resistance, via killing of susceptible microbes. Our work also suggests that restoring a disrupted microbiota after antibiotic treatment (e.g. through microbiota transplants [51] or probiotics [52]) could constrain the competitive advantage, and hence the spread of resistant pathogens. This would rely on monitoring the effects of antimicrobial therapy on not just the pathogen of interest, but the resident microbiota. This can be achieved by, for example, 16S rRNA sequencing [53]. Understanding the effects of antibiotics on the gut microbiota will be of paramount importance in developing new therapeutic strategies to fight against the emergence of resistant pathogens.

## Supplementary data

Supplementary data is available at *EMPH* online.

## Data availability

Datasets are freely available on Dryad: https://doi.org/10.5061/dryad.1rn8pk0tt.

## Supplementary Material

eoab020_Supplementary_Data
