# Supplementary material for: Species interactions drive the spread of ampicillin resistance in human-associated gut microbiota
Source: Evol Med Public Health. 2021 Jun 24;9(1):256–66. doi: 10.1093/emph/eoab020 (PMC8385247; doi:10.1093/emph/eoab020)
Supplement: eoab020_Supplementary_Data [file eoab020_supplementary_data.zip › Supplementary Figures 13.05.docx]

**Supplementary Figures**


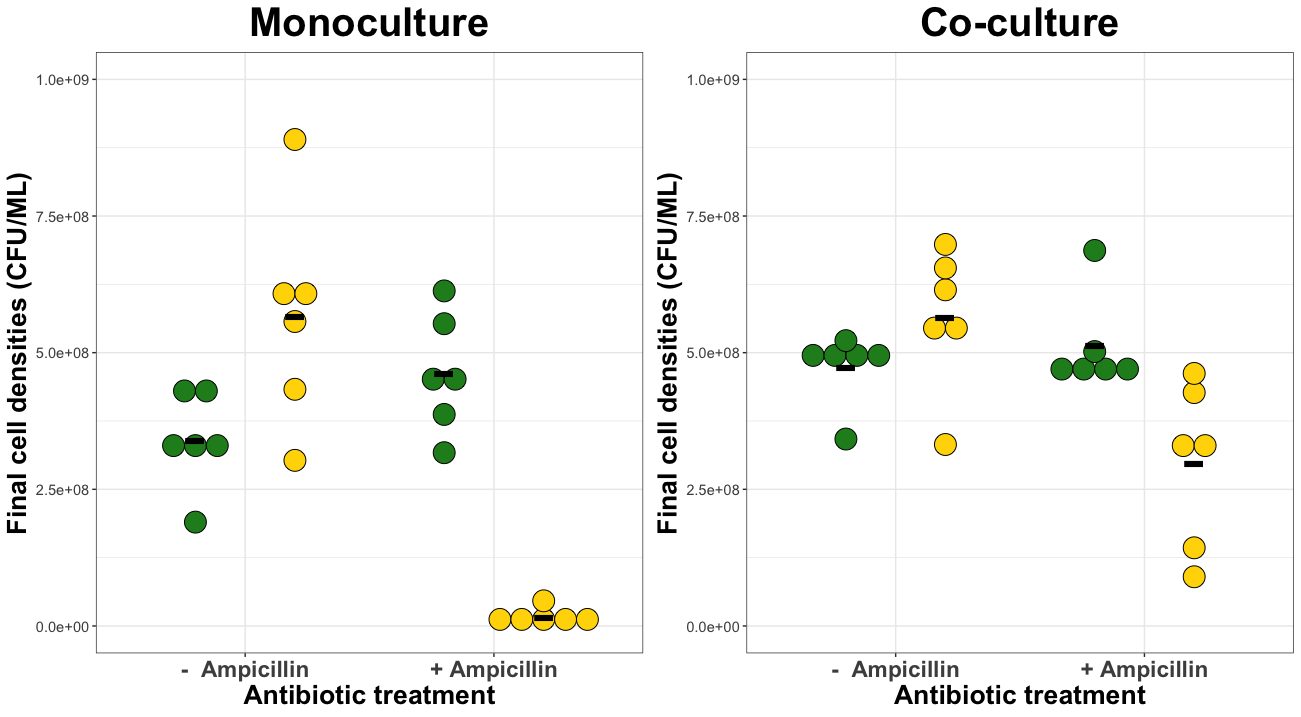


**Figure S1:** Final cell densities (cfu/ml) of susceptible (yellow) and resistant (green) genotypes of our focal *E. coli* strain grown in the presence and absence of ampicillin in monoculture (left panel) or co-culture (right panel) conditions. Ampicillin had a much weaker inhibitory effect on final cell density of K-12_susc_ (yellow points) in co-culture compared to monoculture on average (Linear model; Growth condition × Ampicillin interaction, F_1,20_=6.1223, *p*<0.05). Black horizontal bars show mean values, *n*=6.


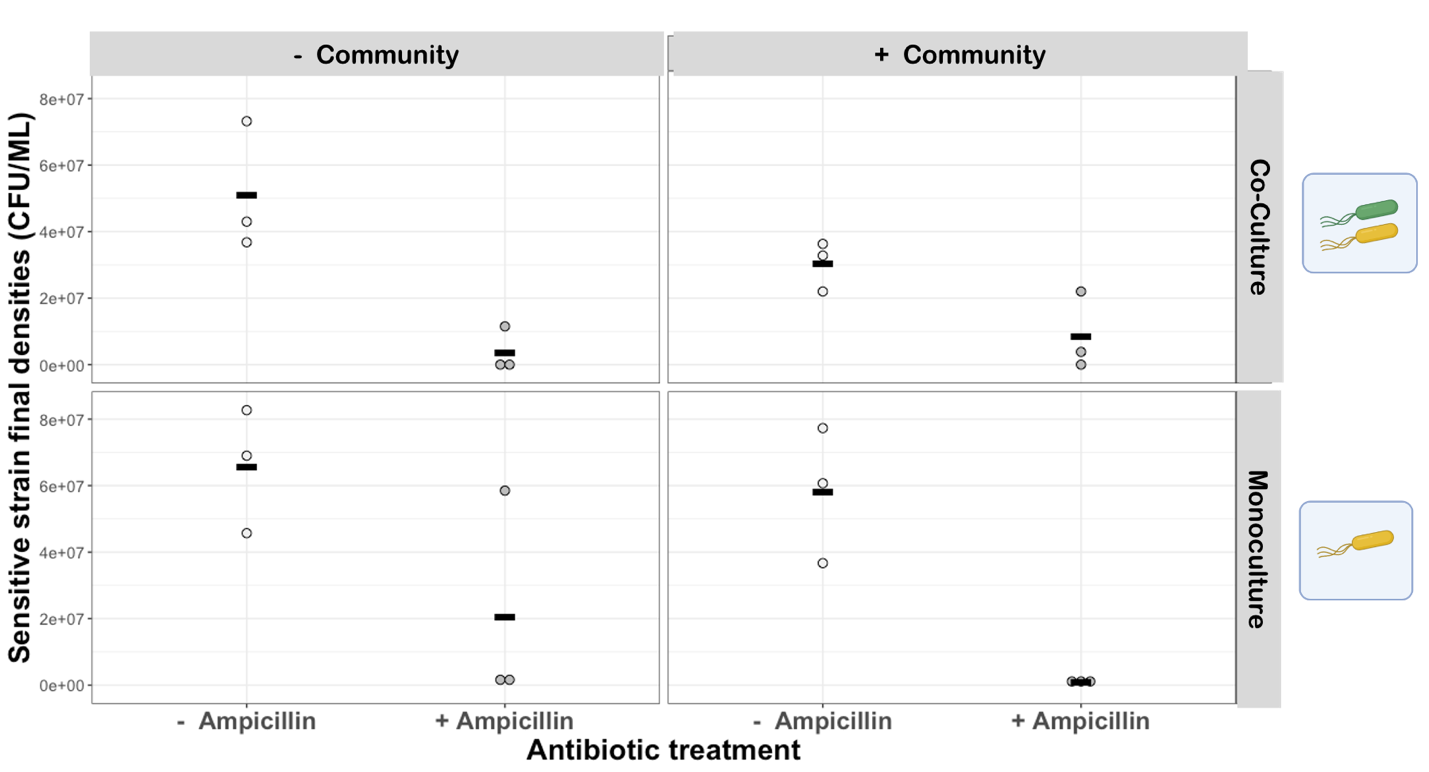


**Figure S2:** Final cell densities (cfu/ml) of K-12_susc_ are unaffected by either the presence of K-12_res_ (mono- versus co-culture) and the presence versus absence of the resident microbial community (Linear model; effect of culture condition, F_1,20_=3.35, *p*=0.08; effect of resident microbiota, F_1,20_=2.32, p=0.14). Ampicillin significantly reduced growth of K-12_susc_, irrespective of culture condition and microbiota presence (Linear model; Significant effect of ampicillin; F_1,20_=36.8, p<0.0001). Hence, we find no evidence that cross protection by resistant microbes reduces the inhibitory effect of ampicillin on K-12_susc_. Black horizontal bars show means, n=3.


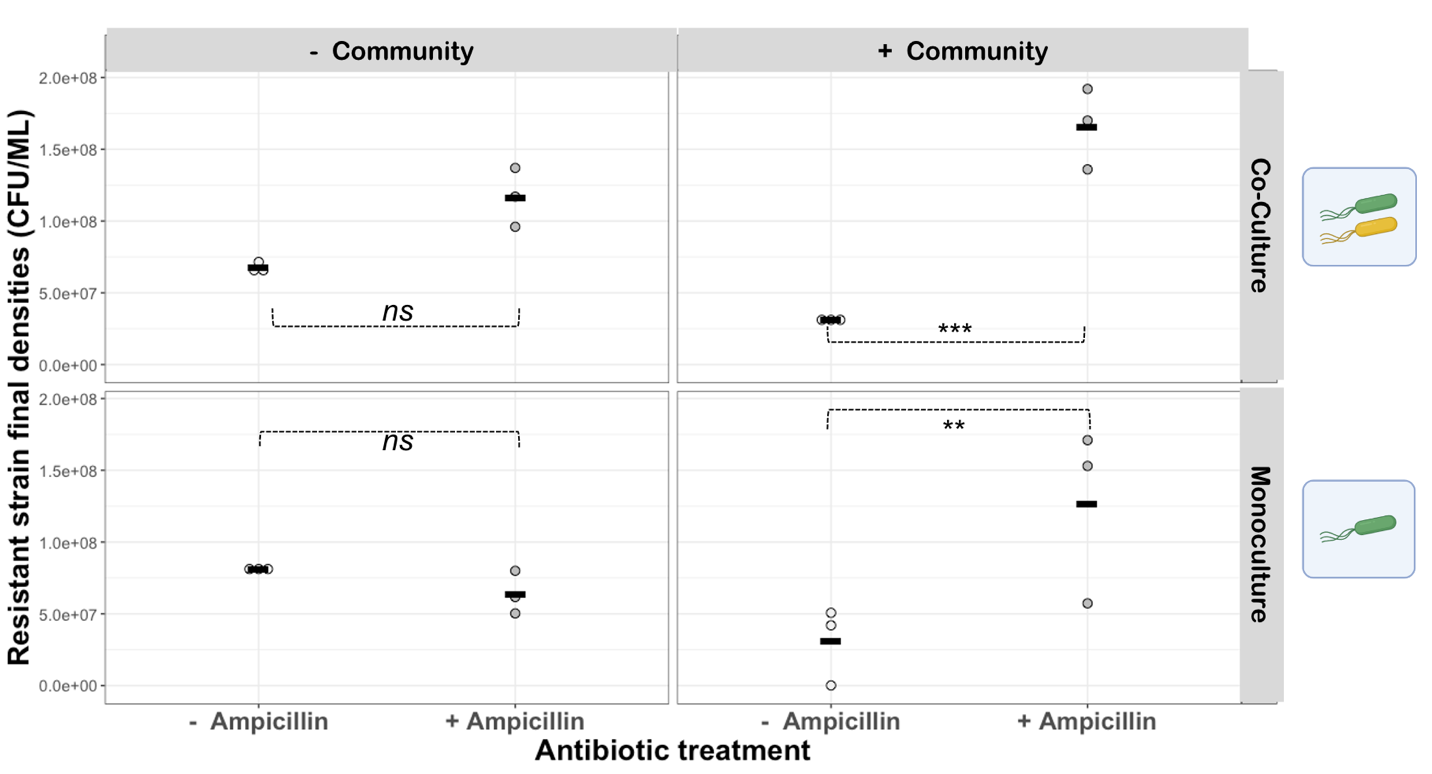


**Figure S3:** Final cell densities (cfu/ml) of K-12_res_ were increased by addition of ampicillin, but only in the presence of the resident microbiota (Linear model; microbiota × ampicillin interaction: F_1,17_=20.78, p<0.001) or K-12_susc_ (Linear model; culture condition × ampicillin interaction: F_1,17_=5.75, p<0.05). This is evidence for antibiotic inhibition of susceptible competitors driving the spread of resistance (competitive release). Black horizontal bars show means, n=3.


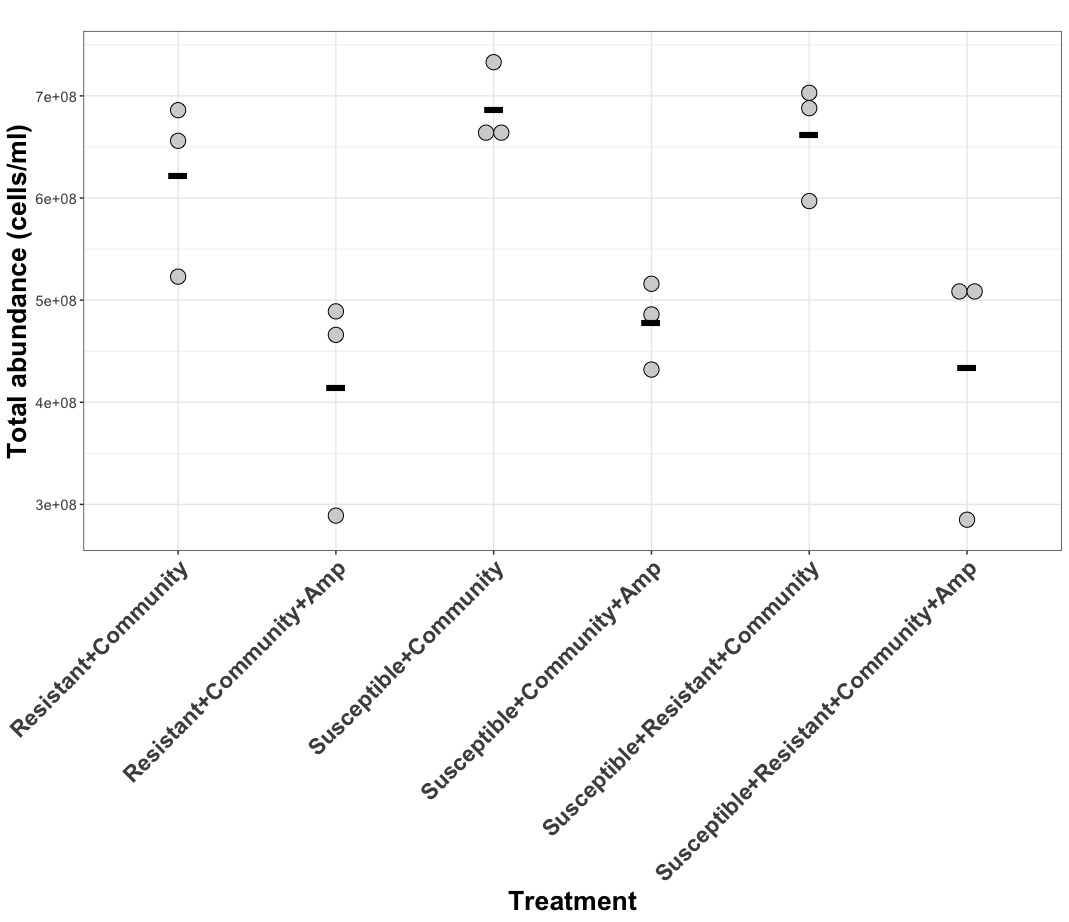


**Figure S4: Total bacterial density (cells/ml) as measured by flow cytometry, in microbial communities in which *E. coli* (K-12_res_, K-12_sus_, or both) is embedded, either with or without antibiotic treatment.** Black horizontal bars show mean values, *n*=3.
