# Supplementary material for: Species interactions drive the spread of ampicillin resistance in human-associated gut microbiota
Source: Evol Med Public Health. 2021 Jun 24;9(1):256–66. doi: 10.1093/emph/eoab020 (PMC8385247; doi:10.1093/emph/eoab020)
Supplement: eoab020_Supplementary_Data [file eoab020_supplementary_data.zip › Supplementary methods S1 04.05.docx]

**Supplementary methods S1**

*Insertion of a Bla_TEM_ plasmid conferring ampicillin resistance into K-12_susc_*

Plasmid DNA was extracted from an *E. coli* strain carrying the pWKS30 plasmid with the ZR plasmid miniprep kit (Zymo) according to manufacturer’s protocol. To generate competent cells of K-12_susc_ for transformation, 500 μl of an overnight culture was inoculated in 20 ml of LB in a 50 ml falcon tube and incubated at 37°C at 180 rpm. On reaching an OD of 0.3 the culture was chilled in an ice-water mix for 5 min before dividing into two 50 ml falcon tubes and centrifuging at 4°C at 4000 g for 5 min. The supernatant was removed, and pellet resuspended in 5ml of 80 mM CaCl_2_ supplemented with 20% glycerol before centrifuging at 4°C at 4000 g for 5 min. This washing step was repeated 3 times. After the last washing step, the pellet was resuspended in 150 μl of the CaCl_2_ glycerol mix and distributed in 1.5 ml Eppendorf tubes in 50 μl aliquots. One of these aliquots was mixed with 1 μl of plasmid extract (50ng of plasmid DNA) and incubated on ice for 30 min. The plasmid-cell mix was then transferred to a preheated 42 °C water bath, incubated for one min and then recovered for 5 min at room temperature. The culture was resuspended in 1 ml of LB and incubated for 30 min at 30 °C before plating on selective plates supplemented with chloramphenicol and ampicillin at 37 °C overnight. Colonies of transformed strains were picked and inoculated in LB supplemented with ampicillin to prepare freezer stocks. Additionally, presence of the plasmid was verified by colony PCR with primers specific for the ampicillin resistance gene (blaFW: 5’-TGCAACTTTATCCGCCTCCA-3’; blaRV 5’-TTGAGAGTTTTCGCCCCGAA-3’).

*Human microbiome sampling regime – outlined fully in [1]*

Samples were collected from consenting, anonymous volunteers at the Department of Environmental Systems Science, ETH Zürich, who were older than 18 years, not obese, and had not taken antibiotics for at least six months. Each sample was collected in a 500ml plastic specimen tube and stored under anaerobic conditions before processing. The faecal slurry of each sample was prepared by suspending 20g of sample into 200ml anaerobic peptone wash (1g/L peptone, 0.5g/L L-Cysteine, 0.5g/L bile salts and 0.001g/L Resazurin; Sigma-Aldrich). Glycerol was added to a final concentration of 20% as a cryoprotective for long-term storage.

**References**

1. Baumgartner M, Bayer F, Pfrunder-Cardozo KR, Buckling A, Hall AR. Resident microbial communities inhibit growth and antibiotic-resistance evolution of Escherichia coli in human gut microbiome samples. PLoS Biol 2020. **18:** e3000465. https://doi.org/10.1371/journal.pbio.3000465.
